# Prevalence and Determinants of Diarrheal Diseases among Under-Five Children in Horo Guduru Wollega Zone, Oromia Region, Western Ethiopia: A Community-Based Cross-Sectional Study

**DOI:** 10.1155/2021/5547742

**Published:** 2021-06-23

**Authors:** Kefalew Alemayehu, Lemessa Oljira, Melake Demena, Abdi Birhanu, Dasselegn Workineh

**Affiliations:** ^1^Guduru Hospital, Kombolcha, Ethiopia; ^2^School of Public Health, College of Health and Medical Sciences, Hararmaya University, Harar, Ethiopia; ^3^Department of Environmental Health Sciences, College of Health and Medical Sciences, Hararmaya University, Harar, Ethiopia; ^4^School of Medicine, College of Health and Medical Sciences, Hararmaya University, Harar, Ethiopia

## Abstract

**Background:**

Diarrheal diseases are the leading cause of preventable death, especially among under-five children in developing countries, including Ethiopia. Although efforts have been made to reduce the morbidity and mortality resulting from diarrheal diseases, there is scarce information on the progress of the interventions against the burdens. Therefore, this study aimed to assess the prevalence of diarrhea and its associated factors in under-five children in Horo Guduru Wollega Zone, Oromia Region, Western Ethiopia.

**Methods:**

A community-based cross-sectional study was conducted. Of 12,316 households, 620 households that had under-five children were selected by simple random sampling technique from randomly selected kebeles. Before data collection, a pretest of the structured questionnaires was done on nonselected kebeles. Binary logistic regression was used to assess the association of the diarrheal diseases with independent variables. Finally, the odds ratio along with a 95% confidence interval was used to report the significant association between the outcome variable and its associated factors. A *P* value of ≤0.05 was considered statistically significant

**Results:**

The prevalence of diarrhea among under-five children was 149 (24%) (95% CI: 20.8, 27.3). Diarrhea was significantly associated with poor knowledge of mothers/caretakers on diarrhea prevention methods (AOR: 2.05, 95% CI (1.14, 3.69), being in the age group of 6–11(AOR = 1.546 (1.68, 3.52), and 12–23 months (AOR = 1.485 (1.84, 2.63)), families with poor wealth index (AOR: 2.41, 95% CI (1.29, 4.51)), children who were not vaccinated against measles (AOR: 4.73, 95% CI (2.43, 9.20)), unsafe child feces disposal (AOR = 3.75; 95% CI (1.91, 7.39)), inappropriate liquid waste disposal (AOR = 3.73 (1.94, 7.42)), and having two or more siblings (AOR: 3.11, 95% CI (1.81, 5.35)). *Conclusion and Remarks.* The prevalence of diarrhea among under-five children was high. There was a statistically significant association between diarrhea and age of the child (6–11 and 12–23), poor knowledge of mothers/caretakers on diarrhea prevention methods, families with poor wealth index, being unvaccinated against measles, improper liquid waste disposal, unsafe child feces disposal, and having at least two siblings. The findings have a significant policy inference for childhood diarrheal disease prevention programs. Therefore, educating mothers/caregivers on diarrheal disease prevention methods, child spacing, regular hand washing practice after disposing child feces, safely disposing liquid waste, and vaccinating all eligible children against measles should be a priority area of intervention for diarrheal disease prevention. Moreover, since these associated factors are preventable, the government needs to strengthen the health extension workers program implementations to reduce childhood diarrhea.

## 1. Introduction

Diarrhea is the passage of unusually loose or watery stools, at least three times in 24 hours. However, it is the consistency of stools rather than the number that is most important. Frequent passing of formed stools is not diarrhea. Babies fed only breast milk often pass loose, “pasty” stools; this is also not diarrhea [1]. There are three main forms of acute childhood diarrhea, all of which are potentially life-threatening and require different treatment courses (acute watery diarrhea, bloody diarrhea, and persistent diarrhea) [2].

Diarrhea is more prevalent in the developing world in the large part due to the lack of safe drinking water, sanitation, and hygiene, as well as poorer overall health and nutritional status. According to the latest available figures, an estimated 2.5 billion people lack improved sanitation facilities, and nearly one billion people do not have access to safe drinking water. These unsanitary environments allow diarrhea-causing pathogens to spread more easily [1].

Globally, there are nearly 1.7 billion cases of childhood diarrheal diseases that account for one in nine child deaths, making diarrhea the second leading cause of death in children under five years old. Even though diarrhea is both preventable and treatable, it kills 525,000 children under five years old each year, and it is a leading cause of malnutrition in children under five years old [3].

The majority (42%) of deaths due to diarrheal disease were concentrated in Sub-Saharan Africa, including Ethiopia (88 per 1000 live births), where hygiene and sanitation are poor [4]. Improved sanitation is one that hygienically separates human excreta from human contact and an improved drinking water source is one that by the nature of its construction adequately protects the source from outside contamination, in particular from fecal matter, and generally, a systematic study conducted in London school of hygiene and tropical medicine revealed that improvement in hygiene especially hand washing with soap alone showed 48% reduction in diarrhea mortality [5]. Even though 63% of the global population use toilet and other improved sanitation facilities, a significant proportion, about 2.6 billion people, lack improved sanitation and 1.1 billion people (15% of the global population) practice open defecation [6].

In Ethiopia, 3/4 of the health problems of under-five children are communicable diseases that come from the environment, especially water and sanitation. Diarrhea is the leading cause of the mortality of under-five children, causing 23% of deaths and around 44% stunting, and in Ethiopia, over 75–80% of communicable diseases are caused due to poor environmental health conditions arising from unsafe and inadequate water supply and poor hygienic and sanitation practices [2]. Nearly two-thirds of the households (65%) obtain their drinking water from improved sources according to the 2016 EDHS report, which declared visible improvement compared with the 2011 EDHS report (54%). The most common source of drinking water in a rural area is a public tab or standpipe (19%), tube well or borehole (13%), and protected spring (14%) [7].

This study considers ODF as a factor that was not considered in all reviewed literatures, except that the sustainability of the program was tested in our country and Asia [8]. Even though the sanitation coverage of the Hababo Guduru District is 76%, open defecation practice is dominant in the district according to the 2009 EC of the district report [9]. Using unsafe drinking water like unprotected springs, wells, rivers, and streams is also the most prominent problem in this area, which is one of the most important causes of diarrheal diseases. Likewise, as far as the investigator's knowledge, no study was conducted in the study area before now. As a result, this study helps to reveal and assess the prevalence and associated factors of diarrhea in under-five children with different demographic and socioeconomic, environmental, and behavioral factors.

## 2. Methods and Materials

### 2.1. Study Design and Setting

A community-based cross-sectional study was conducted from February 15 to March 10, 2018, in Horo Guduru Wollega Zone, Oromia Region, Western Ethiopia, which is located nearly 315 km away from the capital, Addis Ababa. The zone has 12 districts, 11 rural and one urban, and the total population was estimated as 824,205 (male: 412,927 (50.1%); female: 411,278 (49.9%)), and the total numbers of households were 12715 and under-five children in the zone were 123,631.

### 2.2. Population and Eligibility Criteria

All under-five children living in Horo Guduru Wollega Zone were the target population, while children living in randomly selected kebeles in the Horo Guduru Wollega Zone were the study population. Mothers/caregivers-child pairs living in Horo Guduru Wollega Zone for more than six months were eligible for the study.

### 2.3. Sample Size and Sampling Procedures

**Figure 1:**
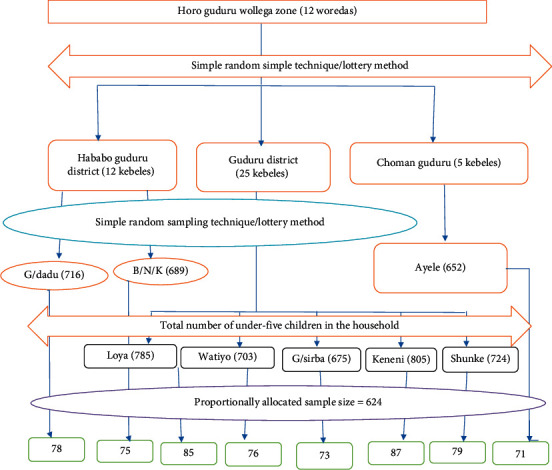
Study participants sampling procedure.

The sample size was determined using EPI-INFO version 7 software programs for double population proportion formula with a 95% level of confidence, 80% power, and design effect of 1.5 and considering the number of under-five siblings [6]. Then, by adding 10% of contingency for the nonresponse rate, the sample size was determined to be 624. A stratified sampling method was employed to select households that had under-five children. Out of twelve districts, three districts were selected by lottery method. By applying the proportional to size allocation method, households with under-five children were selected from each selected kebele. To select each study participant, a simple random sampling technique from the sampled kebeles was used after enumerating the households with under-five children from each of the selected kebeles based on the sample size allocated. Households with at least one under-five child were selected. From the household that has more than one under-five child, only one of the children was randomly selected (Figure 1).

### 2.4. Data Collection Instruments and Procedures

The data were collected by using a structured questionnaire interview. The questionnaires were initially prepared in English and then translated into Afaan Oromoo (the local language of the study area). The data collection questionnaire was developed from the literature review. The adapted questionnaires were modified and contextualized to fit the local situation and the research objective. The data collection was conducted by eight clinical nurses and two BSC holders, which were health officers, assigned as supervisors. A two-day training was given for data collectors and supervisors on how to approach the study groups and fill the questionnaires by the principal investigator. Data collection was performed through house-to-house visits. The data collectors explained the purpose of the study and convinced the study participants to get their consent. For households with two or more children under-five years of age, the candidate child was selected by a lottery method.

### 2.5. Study Variables

The diarrheal disease was an outcome variable of the study independent variables. The sociodemographic and economic variables were as follows: age and sex of the child, mother's education and occupation, and marital status of the mother. Environmental health condition variables were as follows: availability of a toilet facility, type of the toilet, main source of the drinking water, accessibility of drinking water, availability of hand washing facility, hand washing practice, latrine utilization, child's feces disposal practice, water storage practice at home, water treatment practices at home, child feeding practice, measles vaccination status and open defecation field (ODF) status, and knowledge-related variables.

### 2.6. Operational Definitions

  Diarrhea: a child having a history of passing loose stool more than three times per day in the two weeks before the data collection period [10] was considered suffering from diarrhea.
  Narrow-neck storage container: a jerrican was considered a narrow-neck water storage container, while buckets and pots were considered wide-neck water storage containers.
  Knowledgeable: a child's mothers/caretakers who scored above the mean value on answering the knowledge-related questions about diarrheal disease prevention methods were assigned “knowledgeable.”
  Poor knowledge: a child's mothers/caretakers who scored the mean and below the mean value on answering the knowledge-related questions about diarrheal disease prevention methods were assigned “poor knowledge.”

### 2.7. Data Collection Methods and Instruments

Data were collected through house-to-house visits using a structured questionnaire, developed from different kinds of literature. The questionnaires were initially prepared in English and then translated into Afaan Oromoo (the language of the study area). The data collection was conducted by ten clinical nurse professionals and supervised by three public health professionals.

### 2.8. Data Quality Control

The principal investigator provided two days of training for the data collectors and supervisors on the objectives of the study and how to approach the study participants. A pretest was conducted on 5% of study participants living in the nonselected kebeles before the actual data collection period. The data quality was managed at every level of the data collection process. Once the collected data quality consistency and completeness are checked, data were separately entered into EpiData version 3.1 software by two data clerks. Then, data entered into EpiData software were exported to SPSS version 22 for analysis.

### 2.9. Data Analysis

The outcome variable was diarrhea that was dichotomized into having diarrhea in the past two weeks before the data collection period (“Yes” vs “No”). Frequency, proportions, and measures of central tendency were calculated to describe the study subjects. The family wealth index was constructed by using the principal component analysis (PCA) method and considering locally available household assets. Family wealth was categorized into poor, medium, and rich. The multicollinearity effect was tested using the VIF for all independent variables, and no variable was found to have VIF greater than ten. Binary logistic regression was used to assess the associations between dependent and independent variables. Variables that had a *P* < 0.25 in the bivariable analysis were transformed to multivariable analysis. In the multivariable analysis, the adjusted odds ratio (AOR) with its 95% CI was used to determine factors significantly associated with diarrhea in under-five children. A *P* value less than 0.05 was considered statistically significant. The fitness of the model was tested by Hosmer–Lemeshow.

## 3. Results

### 3.1. Sociodemographic Characteristics of Study Participants

A total of 620 households were included in the study with a 99.4% response rate. Three hundred forty-seven (56%) mothers/caretakers were in the age group of 25–34 years, and their mean age and SD was 33 ± 5.54 years. About half of the children (323 (52.1%)) were males. Two hundred seventy-seven (44.7%) children were aged 12–23 months, and their mean age and SD was 20 ± 10.6 months. The majority of mothers (576 (92.6%)) were married at the time of the study, and 212 (34.2%) were unable to read and write. The mean family size was 5.4 ± 1.6 SD. More than one-third of the participants (222 (35.8%)) come from poor families. Concerning the mothers' knowledge on prevention methods of diarrhea in under-five children, the majority (315 (50.8%)) had poor knowledge (Table 1).

### 3.2. Environmental Characteristics

The majority of the households (527 (85.0%)) had a private latrine facility, and more than one-third (348 (1%)) had pit latrines with a slab. Five hundred sixty-three (90.8%) mothers disposed the child's feces into the latrine. About 277 (44.7%) and 379 (61.1%) of the households disposed their solid and liquid wastes everywhere openly, respectively. One hundred seventy (27.1%) and 257 (41.5%) of the households used piped system and protected spring water for drinking purpose, respectively, whereas 331 (53.4%) of the households drunk water without any treatment at their home (Table 2).

### 3.3. Prevalence of Diarrhea

In this study, the prevalence of diarrhea among under-five children was 24% (95% CI: 20.8–27.3). It was slightly higher among rural residents (116 (24.8)) compared with those living in urban areas (33 (21.7)). There was a slight difference in diarrhea prevalence among females (72 (24.2)) and males (77 (23.8)).

### 3.4. Factors Associated with Diarrhea in Under-Five Children

Children whose mothers/caretakers had poor knowledge on diarrhea prevention methods had diarrhea two times more likely than children whose mothers had good knowledge (AOR = 2.05; 95% CI: (1.14, 3.69)). The odds of having diarrhea were 4.7 times more likely in children who were unvaccinated for measles compared with their counterparts (AOR = 4.73; 95% CI: (2.43, 9.20)). The odds of having diarrheal diseases in children whose family inappropriately disposed liquid waste were 3.73 times more likely compared with a family that practiced appropriate disposal of liquid waste (AOR = 3.73; 95% CI: 1.94, 7.42).

Children in the age group of 6–11 months and 12–23 months were 1.5 times (AOR = 1.546; 95% CI: 1.68, 3.52) and 1.4 times (AOR = 1.485; 95%CI: 1.84, 2.63) more likely to have diarrhea than children in the age group of 24 and above months. Children from a family who had two and more siblings were 3.1 times more likely to have diarrhea than children from a family who had only one sibling (AOR = 3.11; 95% CI: 1.81, 5.35). The odds of diarrheal diseases in children whose parents had poor wealth index were 2.41 times more likely compared with children from a family who had a rich wealth index (AOR = 2.41; 95%CI: 1.29, 4.51) (Table 3).

## 4. Discussion

This study uncovered the determinants of diarrhea among children under five years old in Horo Guduru Wollega Zone, Western Ethiopia, which were as follows: children aged 6–11 and 12–23 months, poor knowledge of mothers/caretakers on diarrheal disease prevention methods, children unvaccinated for measles, inappropriate liquid waste disposal system, having more than one child in the family, household with a poor wealth index, and unsanitary feces disposal.

This study examined the sociodemographic and socioeconomic characteristics, feeding and healthcare characteristics, and environmental characteristics associated with diarrheal status in under-five children. The current study showed that, in this study, the prevalence of diarrhea in under-five age children was 24% (95% CI: 20.8–27.3). The prevalence of diarrhea in under-five children in this study was comparable with a study conducted in Cameroon in Sub-Saharan Africa [10] and Eastern Ethiopia (22.5%) [11]. The prevalence of diarrhea in this study was lower compared with cross-sectional studies conducted in Niger (36.4%), North West Burundi (32.6%), and Afar Region (37.5%) [12–14]. However, the finding was relatively high compared with a study conducted in Ghana (13%) [15] and Southern Ethiopia (13.6%) [16]. A discrepancy could be due to the study population difference.

The study showed that diarrhea was significantly associated with children in the age groups 6–11 months and 12–23 months compared with children aged more than or equal to 24 months. This finding is in agreement with other studies [11, 17–19]. Children in this age group may experience mouthing of visibly dirty fomites, feces, soil, and other dirty objects. Therefore, the possible explanation may be due to the mouthing of contaminated objects with diarrhea-causing pathogens, making this group of children at a greater risk of developing diarrheal diseases.

The study revealed that the odds of having diarrhea were higher among families who had at least two siblings compared with those who had only one under-five child. This is in agreement with a study conducted in Cameroon [10], Eastern Ethiopia [11], Uganda [20], and Northern Ethiopia [21]. This might be due to the incapability of parents to provide a balanced diet, safe and clean water, appropriate sanitation service, and early medical care when getting sick.

The study found that the odds of diarrheal diseases in children whose families had a poor wealth index were higher than children whose families were rich. This study's result was in line with a study conducted in Northeast Ethiopia [14]. Being poor may form situations that favor the spread of infectious diseases like diarrhea and limit affected peoples from gaining sufficient access to prevention and care. The possible explanation may be that rich families may have a greater opportunity to use adequate water and soap for hands washing and aqua-guard at their houses to protect against microbial contamination in water, and they may construct a toilet. In addition, the living room and kitchen floor type affects the level of pathogen load that may cause diarrheal diseases. For instance, poor families might construct the floor of the living room or kitchen from mud, which serves as a source of fecal-oral route of transmission of diarrhea causative agents, unlike cemented floors.

Children who were not vaccinated against measles had higher odds of developing diarrheal diseases than their counterparts. This finding is supported by studies conducted in low- and middle-income countries [22] and African and Asian countries [23]. Any child who is not vaccinated against measles has a high probability of developing measles infection. As a result, children may develop measles, which indirectly causes diarrhea as a complication or secondary infection to measles.

Findings on liquid waste disposal showed that children of mothers/caretakers who practiced inappropriate disposal of liquid waste had higher odds of diarrhea compared with children whose mothers/caregivers who practiced proper disposal of liquid waste. This study was consistent with a study from Senegal [24] and Somali Region [25]. This can be explained by the fact that liquid waste contains different diarrheal causing germs, which can be easily distributed by flies. Inappropriate management of the liquid waste pathogens may contaminate foods that the child eats.

Children whose families inappropriately handled feces disposal had higher odds of contracting diarrheal diseases than their counterparts. This was in line with the studies conducted in Benishangul-Gumuz Regional State [19] and Northeast Ethiopia [26]. This can be due to transmission of diarrheal diseases from an infected host to a healthy individual through the contamination of the foods/utensils or drinking water with the infected feces. In addition, both liquid and solid wastes can be the breading sites of insects that serve as a carrier for diarrheal disease causing pathogens.

## 5. Conclusion and Remarks

This study showed the prevalence of childhood diarrhea was high (24%). Children aged 6–11 and 12–23 months, presence of two or more under-five children in the family, mothers/caretakers' knowledge on diarrheal disease prevention methods, inappropriate liquid waste disposal, being unvaccinated against measles, unsafe dispose of feces, and coming from a family with poor wealth index were statistically associated with diarrhea. The findings have a significant policy inference for childhood diarrheal disease intervention programs. Therefore, educating mothers/caregivers on diarrheal disease prevention methods, child spacing, regular hand washing practice after disposing feces, safely disposing liquid waste, and vaccinating all eligible children against measles should be a priority area of intervention for diarrheal disease prevention. Moreover, since these associated factors are preventable, the government needs to strengthen the health extension workers program implementations.

## Figures and Tables

**Table 1:** Sociodemographic characteristics of participants in Horo Guduru Wollega Zone, Oromia Region, Western Ethiopia, from February 15 to March 10, 2018 (*n* = 620).

Characteristics	Categories	Frequency (*n* = 620)	Percentage
Age of mothers/caretakers (years)	<24	31	5.0
	25–34	347	56.0
	≥35	242	39.0

Residence	Rural	468	75.5
	Urban	152	24.5

Ethnicity	Oromo	554	89.4
	Amara	65	10.5
	Gurage	1	0.2

Religion	Muslim	58	9.4
	Orthodox	196	31.6
	Protestant	350	56.5
	Others^*∗*^	16	2.6

Educational status of mothers/caretakers	Unable to read and write	212	34.2
	Informal education	126	20.3
	Grades 1–8	184	29.7
	Grades 9–12	40	6.5
	College and above	58	9.4

Marital status	Single	35	5.6
	Married	576	92.9
	Others^*∗*^^a^	9	1.5

Main occupation of mothers/caretakers	Housewife	362	58.4
	Merchant	183	29.5
	Employee (government/private)	75	12.1

Family size	≤4	190	30.6
	>4	430	69.4

Number of children in the family	≥2	160	25.8
	1	460	74.2

Age of the child in months	0–5	48	7.7
	6–11	71	11.5
	12–23	277	44.7
	>=24	224	36.1

Wealth index	Poor	222	35.8
	Medium	194	31.3
	Rich	204	32.9

Knowledge of mothers/caretakers on diarrhea prevention	Poor	315	50.8
	Good	305	49.2

^*∗*^Wakefata and Catholic. ^*∗*^^a^ Widowed, divorced, or separated.

**Table 2:** Environmental characteristics of participants in Horo Guduru Wollega Zone, Oromia Region, Western Ethiopia, from February 15 to March 10, 2018 (*n* = 620).

Characteristics	Categories	Frequency (*n* = 620)	%
Main source of drinking water	Piped system	170	27.4
	Protected spring	257	41.5
	Well	162	26.1
	River/stream water	31	5

Mechanism of water treatment	Boil	65	10.5
	Add chemical	180	29
	Filter through cloth	43	6.9
	Do nothing	332	53.5

Type of storage container used	Jerrican	498	80.3
	Clay pot	87	14
	Bucket	35	5.6

Type of latrine	Ventilated improved pit latrine	36	5.8
	Pit latrine with slab	348	56.1
	Pit latrine without slab	142	22.9
	Open pit	94	15.2

Child stool disposal	Dispose in the toilet	563	90.8
	Dispose somewhere in the yard	57	9.2

Solid waste disposal method	Dispose openly	273	44.7
	Dispose in a prepared hole	343	55.3
	Dispose in a communal latrine	4	0.6

Liquid waste disposal method	Dispose openly everywhere	379	61.1
	Dispose privately at a designated place	234	37.7
	Dispose in a road ditch	7	1.1

Hand washing mechanism	Without soap/ash	111	17.9
	With soap/ash	509	82.1

ODF status of the kebeles	Yes	319	51.5
	No	301	48.5

**Table 3:** Bivariable and multivariable logistic regressions of factors associated with diarrhea among under-five children in Horo Guduru Wollega Zone, Oromia Region, Western Ethiopia, from February 15 to March 10, 2018.

Variables		Diarrhea status		COR (95% CI)	AOR (95% CI)	*P*
		Yes	No			
		Frequency (%)	Frequency (%)			
Age of child (in months)	<6	6 (12.5)	42 (87.5)	0.619 (0.247, 1.552)	1.066 (0.11, 10.40)	
	6–11	26 (36.6)	45 (63.4)	2.504 (1.391, 4.507)^*∗*^	1.546 (1.68, 3.52)	0.02
	12–23	75 (27.1)	202 (72.9)	1.609 (1.049, 2.467)^*∗*^	1.485 (1.84, 2.63)	0.01
	>=24	42 (18.8)	182 (81.3)	1	1	

Number of children in the family	Two and above	66 (41.3)	94 (58.8)	3.189 (2.150, 4.731)^*∗*^	3.11 (1.81, 5.35)^*∗*^	≤0.001
	One	83 (18)	377 (82)	1	1	

Knowledge on diarrhea prevention methods	Poor	111 (35.2)	204 (64.8)	3.823 (2.535, 5.767)^*∗*^	2.05 (1.14, 3.69)^*∗*^	0.02
	Good	38 (12.5)	267 (87.5)	1	1	

Wealth index	Poor	83 (33.5)	165 (66.5)	3.761 (2.222, 6.365)^*∗*^	2.41 (1.29, 4.51)^*∗*^	0.01
	Medium	45 (23.2)	149 (76.8)	2.258 (1.284, 3.970)^*∗*^	1.06 (0.54, 2.06)	0.9
	Rich	21 (11.8)	157 (88.2)	1	1	

Complementary feeding initiation time (*n* = 569) (months)	<6	52 (42.3)	71 (57.7)	5.696 (2.609, 12.437)^*∗*^	1.46 (0.52, 4.08)	0.47
	>6	83 (22.6)	284 (77.4)	2.273 (1.089, 4.745)^*∗*^	2.08 (0.83, 5.18)	0.12
	At 6	9 (11.4)	70 (88.6)	1	1	

Vaccination status (measles)	Unvaccinated	69 (45.4)	83 (54.6)	4.032 (2.703, 6.01)^*∗*^	4.73 (2.43, 9.20)^*∗*^	≤0.001
	Vaccinated	80 (17.1)	388 (82.9)	1	1	

Solid waste disposal system	Improper	100 (36)	177 (64)	3.390 (2.30, 5.00)^*∗*^	0.85 (0.45, 1.63)	0.63
	Proper	49 (14.3)	294 (85.7)	1	1	

Liquid waste disposal system	Improper	123 (31.9)	263 (68.1)	3.741 (2.361, 5.930)^*∗*^	3.73 (1.94, 7.42)	0.02
	Proper	26 (11.1)	208 (88.9)	1	1	

Child feces disposal system	Unsafe	29 (50.9)	28 (49.1)	3.824 (2.190, 6.674)^*∗*^	3.75 (1.91, 7.39)	0.01
	Safe	120 (21.3)	443 (78.7)	1	1	

Hand washing habit	Using water only	58 (52.7)	52 (47.3)	5.288 (3.401, 8.223)^*∗*^	1.46 (0.69, 3.1)	0.32
	Using ash and water	6 (27.3)	16 (72.7)	1.778 (0.676, 4.676)	1.970 (0.55, 7.14)	0.29
	Using soap and water	85 (17.4)	403 (82.6)	1	1	

Drinking water storage container type	Wide-neck containers	53 (43.4)	69 (56.6)	3.216 (2.11, 4.90)^*∗*^	1.92 (0.9-, 3.57)	0.13
	Jerrican (narrow neck)	96 (19.3)	402 (80.7)	1	1	

Method of cleaning water storage container	Using water	75 (42.4)	102 (57.6)	3.667 (2.485, 5.41)^*∗*^		0.08
	Using soap and water	74 (16.7)	369 (83.3)	1	1	

Significant association = ^*∗*^(*P* < 0.05); COR: crude odds ratio; AOR: adjusted odds ratio; CI: confidence interval.

## Data Availability

The data used to support the study findings are available from the corresponding author upon request.
